# Microenvironment Determinants of Brain Metastasis

**DOI:** 10.1186/2045-3701-1-8

**Published:** 2011-02-25

**Authors:** Chenyu Zhang, Dihua Yu

**Affiliations:** 1 Department of Molecular and Cellular Oncology, The University of Texas M.D. Anderson Cancer Center, Houston, Texas, USA

## Abstract

Metastasis accounts for 90% of cancer-related mortality. Brain metastases generally present during the late stages in the natural history of cancer progression. Recent advances in cancer treatment and management have resulted in better control of systemic disease metastatic to organs other than the brain and improved patient survival. However, patients who experience recurrent disease manifest an increasing number of brain metastases, which are usually refractory to therapies. To meet the new challenges of controlling brain metastasis, the research community has been tackling the problem with novel experimental models and research tools, which have led to an improved understanding of brain metastasis. The time-tested "seed-and-soil" hypothesis of metastasis indicates that successful outgrowth of deadly metastatic tumors depends on permissible interactions between the metastatic cancer cells and the site-specific microenvironment in the host organs. Consistently, recent studies indicate that the brain, the major component of the central nervous system, has unique physiological features that can determine the outcome of metastatic tumor growth. The current review summarizes recent discoveries on these tumor-brain interactions, and the potential clinical implications these novel findings could have for the better treatment of patients with brain metastasis.

## Introduction

Brain metastasis is the major cancerous disease in the central nervous system (CNS), outnumbering primary brain tumor cases 10-fold [1]. Lung cancer, breast cancer and melanoma account for most clinical cases of brain metastasis from non-CNS primary tumors [2]. Brain metastasis often manifests at late stages of metastatic disease progression and causes rapid deterioration in patients' quality of life including neurocognitive impairment [3], although latency varies among different tumor types and many small cell lung cancer patients already exhibit metastatic lesions in the CNS at the time of primary tumor diagnosis. Distinct tumor cell properties from different primary organ sites are likely critical factors responsible for the discrepancy in brain metastasis latency, though the exact molecular mechanism remains elusive. With advances in cancer treatments that better control systemic metastatic diseases at other organ sites, more brain metastasis has emerged in the clinic as exemplified in the cases of HER2-positive breast cancer patients treated by the monoclonal antibody trastuzumab (Herceptin). More than one-third of trastuzumab-treated patients developed brain metastasis in clinical trials [4-6]. Brain metastatic tumors are generally refractory to conventional chemotherapy and the recently developed targeted therapeutic regimens, presumably due to the inability of these therapeutic agents to penetrate the blood-brain barrier (BBB). Current standard treatments for brain metastasis include surgical resection, whole brain radiation therapy (WBRT) or more focused radio-surgical procedures for small numbers of tumor lesions in the CNS [7]. Brain metastasis presents an emerging and urgent unmet medical need and that has been historically understudied. Recently, there has been a steady increase of reports in the literature studying brain metastasis from various primary tumor sites of origin. The current review will emphasize the unique challenges posed by brain metastasis and the latest developments in the field.

## I. Brain Metastasis Models

The metastatic process is a multi-step cascade that requires the completion of a series of highly complex biological functions by tumor cells, including local invasion of the basement membrane, intravasation into the blood vessels, survival in the circulation, extravasation into the target organ tissue and successful colonization in the distant metastatic site [8]. Disruption of any one of these steps would abolish the metastatic process. Hence, a physiologically relevant and reliable model system is essential for the study of metastasis. A conventional experimental metastasis assay uses *in vivo *tail vein injection to achieve hematogenous delivery of tumor cells. However, most tumor cells injected in this way are trapped in the lungs, as they are the first organ encountered with an extensive capillary bed. While large numbers of lung metastases can be reliably produced by tail vein injection, overt brain metastases were rarely developed in these models, partially due to the fact that animals with lung metastases do not survive long enough for brain metastasis to emerge. Alternatively, two other *in vivo *injection routes were developed to produce experimental brain metastasis, both of which target the brain as the first capillary bed that injected tumor cells reach [9]. Direct injection of tumor cells into the left cardiac ventricle is technically easy to perform; the difficulty lies in reliably controlling the exact number of injected tumor cells due to the necessity of maintaining the needle tip steady in a beating heart during the entire injection. Intra-carotid artery injection of tumor cells requires highly sophisticated microsurgical skills but produces experimental results of smaller variation.

Highly organ-specific tumor cell variants, including brain-seeking cell lines, can be selected after repeated application of *in vivo *injections [10,11]. Of particular note, a subline of spontaneous brain metastasis was recently established from *in vivo *recycling of a human melanoma cell line WM239A by subcutaneous implantation and subsequent resection of the primary tumors [12].

The spontaneous metastasis model has the advantage of recapitulating all the important steps of the entire metastatic cascade, providing a unique platform to dissect the molecular mechanisms underlying all of the individual steps of metastasis. Though various human tumor cell lines of different origin have been used to establish brain metastasis mouse models, the pattern of experimental brain metastasis does not seem to reflect the discrepancy of metastasis latency that is observed in the clinic. In spite of this deficiency, the newly established murine models are extremely helpful in dissecting the pathobiology of brain metastasis.

## II. Seed and Soil Hypothesis of Metastasis and the Brain Microenvironment

In 1889, after carefully examining more than 700 cases of breast cancer patients, British surgeon Stephen Paget postulated that cancer metastasis is not a random event and that successful metastatic tumor growth requires compatible interaction between the tumor cells ("seed") and the distant organ tissues to which tumors metastasize ("soil") [13]. In the following 120 years, numerous clinical observations and scientific experiments support the "seed-and-soil" hypothesis, which has become the conceptual foundation for modern studies of cancer metastasis. It is no exception for brain metastasis: unique factors in the brain microenvironment can determine its interaction with metastatic tumor cells and the eventual outgrowth of metastatic tumor lesions.

The brain is the most complex organ, both structurally and physiologically, in the human body, and the site where the majority of CNS functions are performed. There are two major cell types in the brain, neurons and glial cells (astroglia, microglia, oligodendroglia and satellite cells). From a cellular standpoint, the brain is also the most heterogeneous organ in the body since no two neurons are the same and various glial cells serve drastically different functions to maintain brain homeostasis and support neuronal functions. In addition to the parenchymal tissue where all of the neurons and glial cells reside, the brain ventricles form a contiguous compartment with the leptomeningeal space that separates the parenchymal tissue and the skull. Cerebral-spinal fluid (CSF) is produced in the ventricles and flows in this compartment to create a buffer for the entire brain. The leptomeningeal space represents a distinct microenvironment within the CNS and is often another preferred site for clinically-presenting tumor metastasis [14]. However, very few studies have been reported in literature to investigate tumor metastasis to this compartment [15,16], largely due to the lack of appropriate animal models that reflect clinical observations. This review focuses on the microenvironment determinants in the brain parenchyma.

The brain's interaction with metastatic tumor cells largely stems from three unique properties, which include the presence of blood-brain/tumor barriers, large nutrient supply and energy consumption, and its status as a site of immune privilege. Metastatic cells take advantage of these properties for their successful growth in the brain. Recent progress from different fields has shed new light on each of these brain functions and their relationship to metastatic tumor outgrowth.

## III. Blood-Brain/Tumor Barrier and Vascular Re-modeling

The brain has the highest vascular density among all organs, yet the brain endothelia form tight junctions that separate the brain parenchyma from the body's general blood circulation. The circulating small molecules and cells that are easily permeable through the blood vessel wall in other organs are usually prevented from entering the brain parenchyma through the brain endothelia, thus termed the "blood-brain barrier" (BBB). It is generally agreed that such an evolutionary outcome is to protect the brain parenchyma, where essential physiological functions critical for the continuous proper operation of the entire body are performed. Most conventional chemotherapeutic or targeted therapeutic agents cannot penetrate the BBB, thereby posing a tremendous obstacle to the treatment of brain metastatic tumors. On the other hand, precisely how tumor cells are able to penetrate the BBB and gain access to the brain parenchyma has not been studied. Strictly speaking, once tumor cells have outgrown and developed their own blood supply, the interface between the blood vessels within the tumor are termed the "blood-tumor barrier" and should be considered different from the BBB. Recently, state-of-the-art imaging technologies have been used to track isotope-labelled chemotherapeutic drugs and measure BBB permeability simultaneously [17]. It was demonstrated that while some tumor-associated blood vessels may become leaky in the brain, many blood-tumor barriers are impermeable to therapeutic agents used in the study. Furthermore, no significant correlation was observed between the size of the metastatic tumor and the leakiness of the blood vessels within the tumor. The study also discovered an unexpectedly high heterogeneity of the permeabilities of tumor-associated blood vessels. The clinically important implication is that the capability to penetrate the BBB will be essential for any new chemotherapeutic drugs to be successful in treating brain metastasis [18]. Recently, multiphoton microscopy has been used to monitor live tumor cells in the brain for weeks after hematogenous implantation, thus revealing the vascular remodelling processes during early-stage tumor cell colonization [19]. In addition to capturing the live tumor cell extravasation through the BBB, comparison of a pair of lung cancer cells and melanoma cells suggested that the divergent pathways of either co-opted microvascular remodelling and simultaneous growth merely a few days after successful tumor cell extravasation or the slower growth factor-induced angiogenic growth accounts for the length of latency in forming brain metastases. Although the molecular mechanisms underlying these tumor-vessel interactions and their resolution with the clinical observations remain still to be elucidated and determined, the application of these new technologies should greatly help future investigators study these events *in vivo *with unprecedented precision. Adding another twist to the study of these complex interactions, immunohistochemical (IHC) staining of surgically resected breast cancer brain metastases has revealed a more disrupted BBB in brain metastasis from triple-negative/basal-like breast cancers than that from HER2-positive breast cancers [20]. In spite of these exciting studies, the exact molecular mechanisms that regulate the BBB remain unknown. In a recent pair of reports in Nature, experiments using transgenic and knockout mice clearly demonstrated that the pericytes surrounding the brain blood vessels play an intimate role in the formation and maintenance of BBB integrity, challenging the conventional view that astroglial cells play the major role in regulating the BBB [21,22]. Thus, it would be interesting to ask whether alterations of the BBB in brain metastasis are mediated by tumor-pericyte interactions. Unfortunately, no such studies have been reported to date.

Even though the brain has a very dense network of pre-existing vasculature, several groups have reported that the potent angiogenesis/permeability factor, vascular endothelial growth factor (VEGF), seems to be necessary for tumor outgrowth in the brain [23,24]. For example, activation of tumor cell integrin αVβ3 controls brain metastasis through regulation of VEGF expression [25]. Whether tumor-associated blood vessels are formed by new vessel formation or by co-option of existing highly dense vessels may ultimately depend on the context of the specific interaction of tumor cells with their immediate microenvironment. Recently, glioblastoma stem-like cells were observed to be able to differentiate into endothelial cells and provide a blood supply within tumor lesions [26,27]. Though not directly linked to brain metastasis, these striking findings may change the current paradigm of blood vessel formation in tumors, including those in the brain metastasis settings. These novel findings may also impact the therapeutic manipulation of the BBB, which is likely the key in making current therapeutic agents effective in treating brain metastasis.

## IV. Abundant Nutrient Supply and Elevated Energy Metabolism

The dense network of blood vessels in the brain provides it with abundant supply of oxygen and nutrients. The brain has the highest oxygen and glucose consumption rates in the body, and shows strong positive signals in whole-body ^18^F-FDG PET scans under normal physiological states. To elucidate whether this high energy turnover impacts brain metastasis formation, circulating tumor cells isolated from a breast cancer patient were injected to produce experimental brain metastasis [28]. High-throughput proteomic analysis comparing the brain-seeking variants with the parental cells revealed that the brain-derived variants adopted a unique metabolic profile characteristic of elevated expression of various proteins involved in promoting increased energy utilization. In another study, microarray expression profiling was performed to compare laser-captured tumor cells from surgically resected human breast cancer brain metastasis samples. The study revealed that hexokinase 2 (HK2), which mediates the first step in glucose metabolism by phosphorylating glucose to produce glucose-6-phosphate, is a candidate gene up-regulated in brain metastasis samples [29]. Interestingly, HK2 expression level is also elevated in the brain-seeking derivative of the MDA-MB-231 breast cancer cell line compared with the parental cells. Further loss-of-function genetic studies also confirmed that HK2 contributes to the brain metastasis phenotype.

It was first observed by Nobel laureate Otto Warburg that most cancer cells rely on an alternative metabolic strategy through shifting the major output of cellular metabolism to lactate production even under aerobic conditions, an observation aptly termed the "Warburg Effect" [30]. Warburg further postulated that such changes in metabolism underscore the fundamental cause of cancer [31]. Since the brain provides the most abundant nutrient and energy sources in the body, it is conceivable, but still remains to be tested, that tumors endowed with higher efficiency in utilizing the existing metabolic resources would gain an advantage in producing brain metastasis.

## V. Immune-Privileged Sanctuary with Unique Defensive Mechanisms

The brain is one of the few organs that are separated from circulating immune cells, partially due to the presence of the BBB. Though long regarded as an immune-privileged organ, the brain is not immune deficient. Microglia are a specialized population of glial cells that are regarded as the resident macrophages in the brain. Like macrophages, under proper conditions microglia are phagocytic and able to present antigens. Using transgenic mice carrying a GFP reporter protein driven by the CX3CR1 promoter, which is only active in microglia cells within the brain, two groups made the same surprising observation that even under healthy and normal physiological conditions microglia constantly remodel their processes in apparent attempts to survey the brain parenchymal environment [32,33], refuting the conventional view that "resting" microglia are not functionally active.

Astroglial cells, also commonly known as astrocytes, are another major population of glial cells in the brain. They carry out house-keeping functions in the brain to maintain homeostasis and deliver nutrients and energy. Activation of microglial and astroglial cells are found in a wide range of brain-related diseases such as neurodegenerative diseases, traumatic injuries and brain tumors, including both primary and brain metastases. Intensive activation of glial cells is a prominent feature in both patients' brain metastasis tissues and animal models of the disease [34]. IHC staining indicated that even at the early stages of the metastatic cascade shortly after tumor cell extravasation, both microglia and astrocyte cells are activated, indicating that the surveillance system of networked glial cells are very sensitive in detecting microscopic foreign objects such as single metastatic tumor cells [35]. With this in mind, it is critical to know how some tumor cells successfully overcome the glial cell-mediated defensive mechanisms and outgrow to produce overt macro-metastatic lesions. The interactions between tumor cells and glial cells in their immediate microenvironment are poorly studied. One possible mechanism is that tumor cells secrete immunosuppressive factors that would help overcome the surveillance by the glial cells [16]. Brain tissue-conditioned medium can also promote growth and soft agar colony formation of brain-seeking variant cells but not the parental cells [34]. Interestingly, astrocytes were shown to protect human melanoma cells from apoptosis by sequestering calcium through physical contact gap junctions in *in vitro *co-culture experiments [36]. A similar *in vitro *experiment indicated that human lung cancer cells and co-cultured mouse astrocytes can engage in a mutual stimulation to promote proliferation, likely through paracrine cytokine signaling [37]. Along this line, tumor cell expression of neurotrophins, a family of proteins involved in neuronal survival and development, was shown to play an important role in promoting melanoma brain metastasis by up-regulating the ECM-degradative enzymes such as heparanase [38].

Although the activation of glial cells in various CNS-related diseases was widely observed, the exact roles they play in disease progression remain elusive and conflicting in the literature. Some studies show that glial activation has neuroprotective effects, whereas others argue that they promote disease progression [39]. Similarly, it is not yet clear whether the activation of glial cells promotes or inhibits brain metastasis *in vivo*. Conclusive demonstration of the functional roles of the glial cells must employ novel animal models where the activation of glial cells can be experimentally manipulated and monitored.

## Conclusions

Brain metastasis has become an increasingly challenging clinical problem, largely due to the recently improved clinical control of systemic metastatic diseases, exemplified by the use of trastuzumab (Herceptin) for HER2-positive breast cancer. Yet the biology of brain metastasis is still poorly understood. It is encouraging to see more efforts are beginning to be directed toward the study of brain metastasis and that an increasing number of new *in vivo *models have emerged. It is undisputable that the microenvironment cells in the tumor stroma contribute significantly to the outgrowth of cancer cells both at the primary site and in distant metastatic organs. The occurrence of brain metastasis reflects the culmination of such tumor-microenvironment interactions. Particularly, the aforementioned specialized physiology of the brain not only contributes to the colonization of metastatic tumor lesions but also significantly affects the efficacy and outcome of therapeutic interventions. In this review, we highlighted three prominent physiological features of the brain that may impact the natural history of tumor cells in the brain. Future clinical interventions to treat patients with brain metastasis must take into consideration the impact of these important microenvironmental determinants.

## List of abbreviations

CNS: central nervous system; BBB: blood-brain barrier; WBRT: whole-brain radiation therapy; CSF: cerebrospinal fluid; IHC: Immunohistochemistry; VEGF: vascular endothelial growth factor

## Competing interests

The authors declare that they have no competing interests.

## Authors' contributions

CZ and DY planned the manuscript outline. CZ wrote the draft manuscript. DY revised and finalized the manuscript. All authors read and approve the final manuscript.
